# Cardiac Function in a Long-Term Follow-Up Study of Moderate and Severe Porcine Model of Chronic Myocardial Infarction

**DOI:** 10.1155/2015/209315

**Published:** 2015-02-23

**Authors:** Renate de Jong, Gerardus P. J. van Hout, Jaco H. Houtgraaf, S. Takashima, Gerard Pasterkamp, Imo Hoefer, Henricus J. Duckers

**Affiliations:** ^1^Molecular Cardiology Laboratory, Erasmus University Medical Center, Room 2389a, P.O. Box 2040, 3000 CA Rotterdam, Netherlands; ^2^Experimental Cardiology Laboratory, University Medical Center Utrecht, Heidelberglaan 100, 3584 CX Utrecht, Netherlands; ^3^Interventional Cardiology Department, Division of Cardiology & Pulmonology, University Medical Center Utrecht, Room E04-201, Heidelberglaan 100, 3584 CX Utrecht, Netherlands

## Abstract

*Background*. Novel therapies need to be evaluated in a relevant large animal model that mimics the clinical course and treatment in a reasonable time frame. To reliably assess therapeutic efficacy, knowledge regarding the translational model and the course of disease is needed. *Methods*. Landrace pigs were subjected to a transient occlusion of the proximal left circumflex artery (LCx) (*n* = 6) or mid-left anterior descending artery (LAD) (*n* = 6) for 150 min. Cardiac function was evaluated before by 2D echocardiography or 3D echocardiography and pressure-volume loop analysis. At 12 weeks of follow-up the heart was excised for histological analysis and infarct size calculations. *Results*. Directly following AMI, LVEF was severely reduced compared to baseline in the LAD group (−17.1 ± 1.6%, *P* = 0.009) compared to only a moderate reduction in the LCx group (−5.9 ± 1.5%, *P* = 0.02) and this effect remained unchanged during 12 weeks of follow-up. *Conclusion*. Two models of chronic MI, representative for different patient groups, can reproducibly be created through clinically relevant ischemia-reperfusion of the mid-LAD and proximal LCx.

## 1. Introduction

The treatment of patients suffering from myocardial infarction (MI) is aimed at the preservation of cardiac function. Most treatment regimens directly target pathways that limit infarct size or reduce adverse remodeling, thereby preventing progression into heart failure (HF) [1–3]. To fully determine the possible effect of such therapies, thorough testing in clinically relevant animal models is needed [4, 5]. Since large animal models enable clinical treatment regimens, delivery route, and identical function-related measurements, they are considered to withhold greater translational value than small animal models and are therefore superior for efficacy testing [6–10].

Importantly, due to optimized logistical and diagnostic health care, the complaint-to-needle time has decreased considerably in the western world [11]. This has resulted in a large proportion of patients with a relatively preserved left ventricular function after MI [12]. These patients could, however, still benefit from therapy that further confines the amount of damage directly after MI. To test the efficacy of such therapies, an animal model that closely resembles the clinical course of disease in a mildly damaged heart is mandatory. At the same time, therapy optimization has also resulted in an increased incidence of HF, since patients survive with severely deteriorated cardiac function [13]. This patient group would greatly benefit from therapy that improves cardiac function. For this purpose, an animal model that closely resembles the progression into HF after MI in patients with a severely damaged heart is needed.

To create both a moderate and a severe model of chronic myocardial infarction that resemble these different patient groups, ischemia can be induced in several ways. These include permanent ligation, progressing occlusion by placement of ameroid constrictors, a bottleneck stent model, and coiling or infusion of ethanol in the target coronary artery [14–18]. However, most of these models do not mimic current clinical reality, where most patients are revascularized within relatively short time after occlusion. Since revascularization of the target vessel remains the cornerstone treatment in MI patients, animal models have to simulate this situation, even more so since myocardial wound healing and other molecular mechanisms are substantially different after revascularization compared to those during persistent ischemia [19, 20]. Moreover, the chronic coronary occlusion precludes intracoronary infusions, the easiest and fastest technique for myocardial delivery of therapeutics. Hence, models of ischemia/reperfusion, in which coronary flow is restored after an ischemic period, seem most suited to validate novel therapies for the treatment of MI.

In most ischemia/reperfusion models, the left anterior descending artery (LAD) is occluded, which is associated with a high mortality rate during infarct induction and reperfusion due to ventricular fibrillation (VF) [8, 21]. As an alternative for LAD occlusion, the left circumflex artery (LCx) can be occluded. The LCx is responsible for approximately 20% of the blood supply to the left ventricle and an occlusion-reperfusion model of this artery may result in a lower mortality rate [21]. However, it is not fully elucidated to what extent animals that are subjected to LCx ischemia-reperfusion develop cardiac dysfunction during long-term follow-up. Moreover, no thorough sequential investigation of cardiac (dys) function and ventricular dilatation has been performed in either model to the best of our knowledge. Therefore, this study was designed to investigate the long-term effect of myocardial infarction on cardiac function in two clinically relevant large animal models of MI: a severe mid-LAD ischemia-reperfusion model and a moderate proximal LCx ischemia-reperfusion model. In the present study we investigated the course of both global and regional cardiac function decay in the two models combined with mortality, infarct size, and histological analysis.

## 2. Objectives

To determine the long-term course of cardiac function in a moderate LCx and a severe LAD ischemia-reperfusion model for future validation of novel therapeutic strategies, we hypothesized that LAD ischemia-reperfusion will result in fast development of cardiac dysfunction representing a good model for severely affected patients with cardiac dilatation and signs of heart failure. Secondly, we hypothesized that the LCx ischemia-reperfusion model leads to a moderately affected heart representing a large patient group that has been adequately revascularized after an initial ischemic event but could still benefit from additional therapy.

## 3. Material and Methods

All procedures were approved by the local animal welfare committee of the University of Utrecht (Utrecht, The Netherlands; Permit number 2011.II.04.068). A total of 12 female specific pathogen free (SPF) landrace pigs were included (65.1 ± 1.0 kg; van Beek, Lelystad, The Netherlands). The pigs were housed in the experimental animal facility at the University of Utrecht (Utrecht, The Netherlands) in a group prior to procedure and individually after the AMI (to prevent hostile behavior after infarct procedure). The animals were checked by a veterinarian upon arrival at the facility and daily scored by a biotechnician and once a week by a veterinarian during follow-up. Six animals were randomized to undergo ischemia-reperfusion of the LCx and 6 animals were randomized to undergo LAD ischemia-reperfusion.

### 3.1. Medical Treatment before Infarct Induction

All animals received dual antiplatelet therapy (acetylsalicylic acid 80 mg/d: Ratiopharm, Haarlem, The Netherlands; clopidogrel 75 mg/d: Apothecon, Barneveld, The Netherlands) and antiarrhythmic drugs (Amiodarone, 1200 mg loading dose, 800 mg qd, Sanofi-Aventis, Paris, France) starting 10 days prior to infarct induction up until sacrifice at 12 weeks of FU.

### 3.2. Anesthesia Protocol

General anesthesia was induced with an intramuscular injection of 0.5 mg/kg midazolam (Actavis, Zug, Switzerland), 10 mg/kg ketamine (Narketan, Vétoquinol, Lure Cedex, France), and 1 mg of atropine (Pharmachemie BV, The Netherlands) and maintained with intravenous infusion of midazolam 0.5 mg/kg/h, sufentanil 2.5 *µ*g/kg/h (Janssen-Cilag BV, Tilburg, The Netherlands), and pancuronium 0.1 mg/kg/h (Inresa, Battenheim, Germany). Upon infarct induction, all animals were therapeutically heparinized with 2 doses of 5000 IE (Leo Pharma, Ballerup, Denmark). All pigs received a fentanyl patch (25 mg, Janssen-Cilag, Tilburg, The Netherlands) and meloxicam (Boehringer-Ingelheim, Alkmaar, The Netherlands) 0.5 mg/kg/d, as postsurgery analgesia.

### 3.3. Infarct Procedure

Animals were randomized before the start of the procedure via sealed envelopes. Cardiac function at baseline was quantified by 2D echocardiography and pressure-volume (PV) loop analysis. Blood was sampled before AMI and 6 hours after for troponin I analysis. All animals received a Reveal event recorder (Medtronic, Tilburg, The Netherlands).

### 3.4. Balloon Occlusion of the LCx

**Figure 1:**
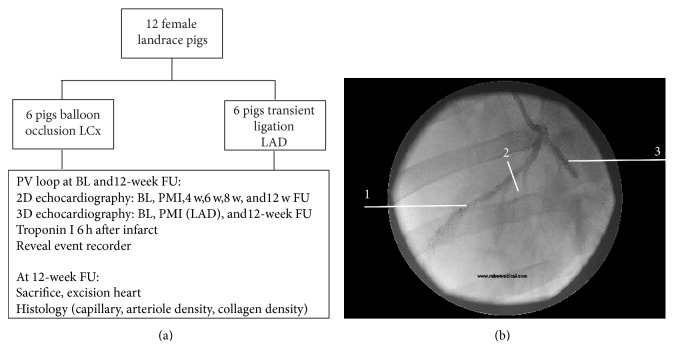
Study design. (a) Flowchart. (b) 1: left anterior descending artery (LAD); 2: ligation of the LAD was located after the first diagonal branch; 3: location and example of balloon occlusion of the left circumflex artery (LCx). BL: baseline before infarct induction; PMI: postmyocardial infarct; w: week.

An 8F sheath was inserted in the carotid artery and an 8F guiding catheter (JL 3.5–4.0, Boston Scientific Nederland BV, Nieuwegein, The Netherlands) was positioned at the ostium of the left main coronary artery. An angioplasty balloon (Trek 3.5–4.0 ×12, Abbott) was inflated (8–14 bar) for 150 min in the proximal LCx (Figure 1). After balloon inflation, the guiding catheter was carefully retracted to enable normal blood flow through the nonoccluded part of the left coronary system. Position was verified every 15 minutes to ensure appropriate occlusion of the LCx. After the procedure catheters were removed and the wound was closed.

### 3.5. Open Chest Ligation of the LAD

An anteroseptal myocardial infarct was induced during an open chest procedure in order to reduce periprocedural mortality due to VF. The thorax was opened via sternotomy. The infarct was induced by a transient ligation of the mid-LAD after the first diagonal for 150 min. All pigs subjected to LAD ischemia-reperfusion underwent a 3D epicardial echocardiography before and after infarct induction.

### 3.6. Follow-Up

During the 12 weeks of FU, a 2D echocardiography was performed at 4 and 8 weeks of FU under induction medication as described above. Twelve weeks after infarct induction the animals were anesthetized, and 2D and 3D epicardial echocardiography were performed followed by invasive PV loop measurements. The animals were sacrificed and the hearts were excised for infarct size determination and histological analyses.

### 3.7. Echocardiography

Echocardiography was performed using a Phillips iE33 echocardiography machine (Phillips, Eindhoven, The Netherlands). 2D images were obtained of the parasternal long axis and parasternal short axis at basal, mid-ventricular, and apical level. The 2D echo was repeated after infarct induction in the animals with LCx infarction. This was not possible in the LAD group because of fluid and air in the chest after open chest procedure. Additionally, a 3D epicardial echocardiogram was performed in the LAD ischemia-reperfusion before and after myocardial infarction as previously described [22]. In both groups, animals underwent additional 2D echocardiography at 4 and 8 weeks following infarct induction, using a mild sedation of midazolam, ketamine, and atropine as described earlier. At sacrifice, both animals in the LCx and LAD group underwent transthoracic 2D and epicardial 3D echocardiography.

Images were analyzed using Velocity Vector Imaging (VVI, Siemens Medical solutions, USA). The end-diastolic and end-systolic volumes were calculated by the modified Simpson rule (LV end-diastolic volume ((LVEDV) = (*A*_*b*ED_)∗*L*/3 + (*A*_*m*ED_ + *A*_*p*ED_)/2)∗*L*/3 + 1/3(*A*_*p*ED_)∗*L*/3; LV end-systolic volume ((LVESV) = (*A*_*b*ES_)∗*L*/3 + (*A*_*m*ES_ + *A*_*p*ES_)/2)∗*L*/3 + 1/3(*A*_*p*ES_)∗*L*/3, in which *A*_*b*_ is the area at basal level, whereas *A*_*m*_ and *A*_*p*_ are the areas at mid and apical levels, resp, and *L* is the length of the ventricle) [23]. LVEF was calculated by ((LVEDV − LVESV)/LVEDV)∗100.

3D echocardiographs were analyzed offline using Qlab 10.1 software (Phillips, Eindhoven, The Netherlands) as described before [24]. One full volume analysis was used and the end-diastolic frame was selected. Markers were placed at the base of anterior, posterior, inferior, and lateral sides and at the apex. The left ventricle was automatically traced by the software. All frames were checked for correct tracing and manually corrected if needed. The same was repeated for the end-systolic phase.

### 3.8. Strain Analysis

**Figure 2:**
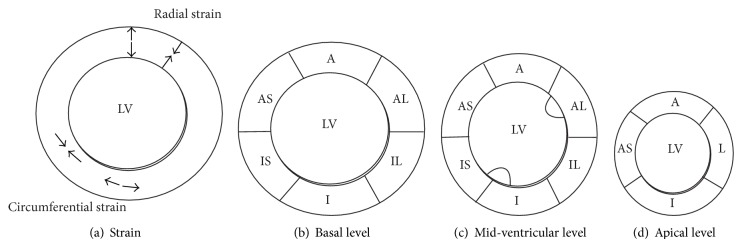
Schematic overview of strain and 17-segment echocardiography model. (a) Schematic overview of strain analysis. Radial strain represents thickening (systole, arrows outwards) and thinning (diastole, arrows facing each other) of the myocardium during 1 cardiac cycle. Circumferential strain: elongation and shortening (arrows facing each other) of myocardial muscle fibers. (b) Schematic overview of myocardial segments on short axis view at basal level (mitral valve level). A indicates anterior wall. AL: anterolateral wall; IL: inferolateral; I: inferior; IS: inferoseptal; AS: anteroseptal. (c) Schematic overview of short axis view of myocardial segments at mid-ventricular level (papillary muscle level). (d) Schematic view of the apex. The apex only consists of 4 segments.* L* indicates lateral wall.

Strain is defined as the total deformation of the myocardium during 1 cardiac cycle [25, 26]. Radial and circumferential strains were analyzed on the 2D echocardiography short axis views via speckle tracking (VVI, Siemens Medical solutions, USA) as previously described [25]. Strain was analyzed according to the 17-segment echocardiography model.  Figure 2 provides a schematic overview of the radial strain and circumferential strain and the 17-segment echocardiography model. In the LAD model anterior and anteroseptal segments were analyzed, whereas the inferior and inferolateral segments were used as reference segments in this group. In the LCx group, inferior and inferolateral segments were mostly affected and anterior segments were used as reference segments. Moreover, global strain was calculated by the software. Strain is represented as percentage of left ventricular deformation.

### 3.9. Pressure-Volume Loop Analysis

A 7F conductance catheter (CD Leycom, Zoetermeer, The Netherlands) was placed under fluoroscopic guidance in the apex of the left ventricle as previously described [27]. Pressure-volume loops (PV loops) were assessed during apnea to avoid pressure/volume changes due to mechanical ventilation. Calibration was performed as previously described [28]. PV loops were performed to obtain data of systolic indices (end-systolic pressure, stroke volume, end-systolic pressure volume relations, stroke work, *dP*/*dt* max, and prerecruitable stroke work) and diastolic indices (end-diastolic pressure, Tau, *dP*/*dt* min, and end-diastolic pressure volume relations). Analyses were performed using Conduct NT analyses software version 16.1 (CD Leycom, Zoetermeer, The Netherlands).

### 3.10. Troponin I Levels

Troponin I levels were quantified using a standardized ELISA protocol of the clinical chemistry laboratory of the University of Utrecht.

### 3.11. Infarct Size Calculations and Histological Analysis

After the heart was excised, the atria were removed and the ventricles were cut into 5 slices of approximately 1 cm thickness. The heart was stained using 5% tetrazolium chloride solution for 10–15 minutes at 37°. The slices were photographed and infarct size was calculated using automatic computer assisted image analysis software (Clemex, Quebec, Canada) as described before [29]. Infarct size was calculated as percentage of the total LV area.

Biopsies were taken from the infarct area, the infarct border zone, and the remote myocardial segments followed by embedding into paraffin for further light microscopical analysis. Collagen content in infarct, border, and remote myocardial segments was assessed by trichrome stain. Briefly, all sections were deparaffinised and fixed in Bouin's fixative (Sigma-Aldrich, St. Louis, USA) at 56° for 15 minutes. Nuclei were stained with haematoxylin for 3 minutes. The slides were submerged in Trichrome-AB solution for 5 minutes after which they were treated with 0.5% acetic acid for 1 minute. Slides were mounted with Entallan (Merck, Darmstadt, Germany). Three random pictures were made at a 10X magnification and collagen content was calculated as percentage collagen of total surface area using automated analysis software (Clemex, Quebec, Canada).

Arteriole density was quantified in infarct, border, and remote myocardial segments, using alpha smooth muscle actin immunohistochemistry analysis (SMA, clone 1a4, Sigma-Aldrich, St. Louis, USA). Endogenous peroxidase activity was blocked by 3% methanol/H_2_O_2_ solution for 30% and incubated with SMA 1 : 1000 overnight. Subsequently, the slides were incubated with secondary HRP-conjugated goat anti-mouse antibody dilution 1 : 200 (DAKO, Glostrup, Denmark) for 90%. All slides were immersed in DAB solution for 2 minutes (DAKO, Glostrup, Denmark) and mounted with Entallan. A technician that was blinded for group allocation took 3 random pictures at 10 times magnification. Arterioles per field of view were counted and expressed as number per view.

Capillary density was only assessed in border in and remote areas, because almost all capillaries are destroyed after AMI. All sections were deparaffinised and pretreated with trypsin EDTA (Lonza, Verviers, Belgium). Endogenous peroxidase activity was blocked as described above. All slides were incubated with Isolectin B_4_ (*Bandeiraea simplicifolia* Isolectin B_4_, Dako, Glostrup, Denmark diluted 1/50) overnight. Subsequently, the slides were immersed in DAB solution and mounted with Entallan. Photographs were taken at 20X magnification and number of capillaries per mm^2^ was calculated.

### 3.12. Statistical Analysis

All data were analyzed using SPSS Statistics 20 (IBM statistics, Chicago, USA). All data are presented as mean ± SEM. Comparisons between groups at a single time point (histology, infarct size, comparison of 12 weeks of FU data) were analyzed using Student's* t*-tests or Mann-Whitney* U* tests. For sequential data, a two-way repeated measures ANOVA was applied. Correlations were analyzed by Pearson's correlation coefficient. *P* < 0.05 was considered statistically significant.

## 4. Results

### 4.1. Mortality and Safety

All animals that underwent LCx occlusion survived initial infarct induction (100%) compared to 5/6 animals in the LAD group (83%; *P* = NS). This animal died of ventricular fibrillation during infarct induction, resistant to defibrillation. Fifty percent of the animals in the LCx group needed defibrillation during infarct induction with an average of 7.8 times, as compared to 5/6 animals in the LAD group with an average of 12.2 times defibrillation. During the 12 weeks of FU period, none of the animals died or were treated for heart failure. No ventricular arrhythmias were recorded on the Reveal detector during the 12 weeks of FU.

### 4.2. Cardiac Function by Echocardiography

**Figure 3:**
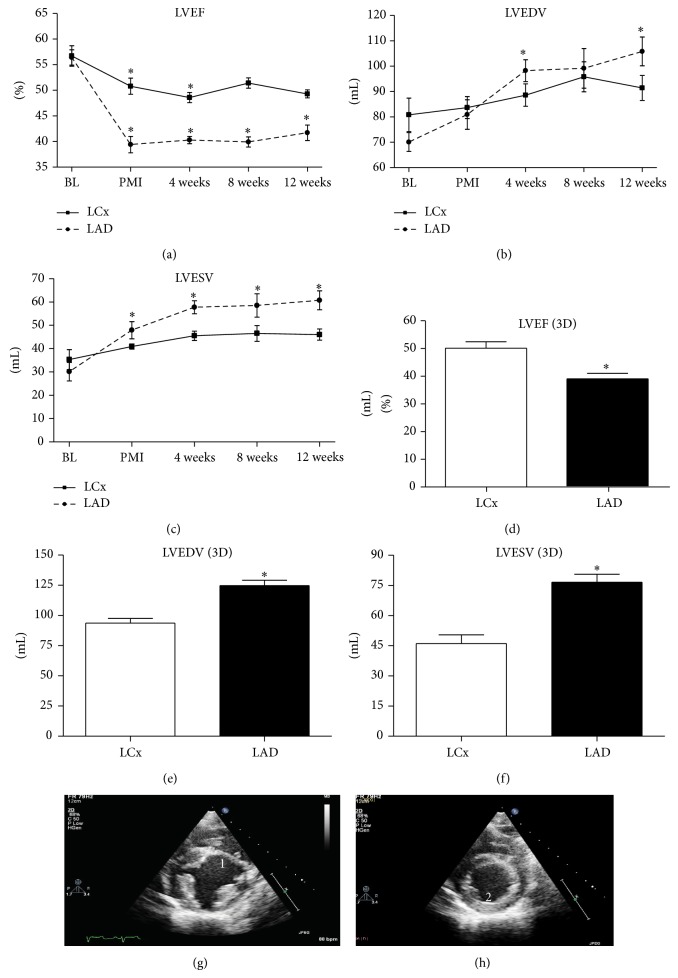
Echocardiography. (a)–(c) LVEF and LV volumes on echocardiography over time. (a) Left ventricular ejection fraction (LVEF) over time. (b) Left ventricular end-diastolic volume (LVEDV) over time. (c) Left ventricular end-systolic volume (LVESV) over time. (d)–(f) 3D echocardiography at 12 weeks of FU. (d) LVEF; (e) LVEDV; and (f) LVESV at 12 weeks of FU as measured by 3D echocardiography. LCx indicates left circumflex artery. LAD: left anterior descending artery. (g) Example of echocardiogram at end of systole at mid-ventricular level at 12 weeks of FU. 1 indicates a formation of scar tissue at 12 week FU. Moreover, the end-systolic diameter has been increased. (h) Example of an echocardiogram of an animal in the LCx group at 12 weeks of FU at mid-ventricular level at the end of systole. 2 indicates the location of the scar. No clear myocardial thinning could be observed in the Lcx model. ^*^ *P* < 0.05.

Both LVEF and LV volumes before infarct induction were comparable between the LCx and LAD group. Post-AMI LVEF decreased with 5.9% to 50.8 ± 1.6% (*P* = 0.03) in the LCx group and with 17.1% to an average of 39.4 ± 1.6% in the LAD group (*P* = 0.009). Following AMI, LVESV increased with 5.6 ± 2.0 mL (*P* = NS) in the LCx group and with 17.8 ± 6.7 mL (*P* = 0.01) in the LAD group (Figure 3). There was no significant change in LVEDV following infarct induction in either of the groups.  Table 1 depicts all cardiac dimensions in both groups.

At 12 weeks of FU, 2D echocardiography revealed a decreased LVEF of 7.9% to 49.3 ± 0.8% (*P* = 0.06) in the LCx group whereas LVEF of the LAD group declined with 14.7% to 41.7 ± 1.5% (*P* < 0.001). LVESV increased nonsignificantly in the LCx group with 10.7 mL to 46.0 ± 2.4 mL (*P* = 0.06), where the LAD group did show a significant increase of 30.6 mL to 60.7 ± 4.1 mL (*P* < 0.001). No significant dilatation as measured by LVEDV was seen in the LCx group (+10.4 mL to 91.4 ± 4.9 mL, *P* = 0.09). LVEDV in pigs subjected to LAD occlusion, however, was significantly increased during follow-up (+35.8 mL to 104.1 ± 5.7 mL, *P* = 0.02). At 12 weeks of FU, 3D echocardiography confirmed the 2D echocardiography measurements (Figure 3). In the LCx group, LVEF was 50.0 ± 2.4% as opposed to 39.2 ± 1.9% in the LAD group. LVEDV in the LCx group at 12 weeks of FU was 98.5 ± 2.9 mL as compared to 126.1 ± 3.5 mL in the LAD group.

### 4.3. Radial and Circumferential Strain

**Figure 4:**
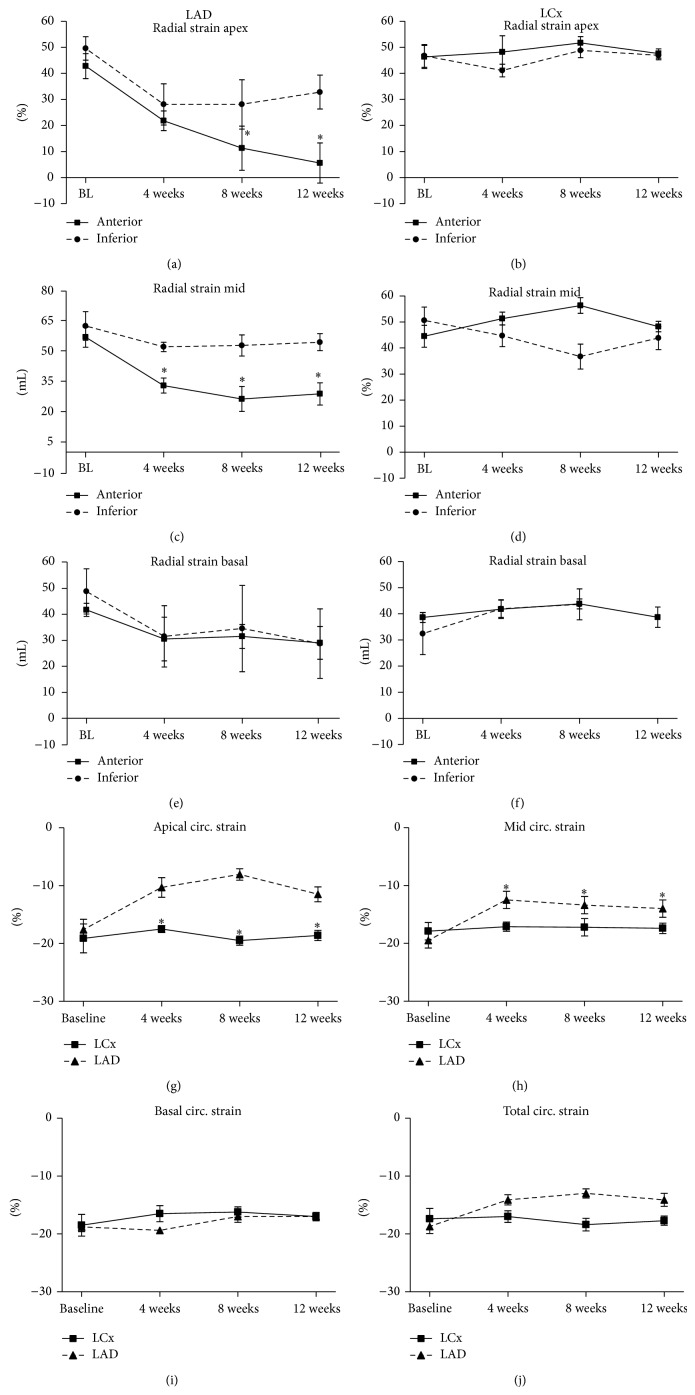
Radial strain and circumferential strain. (a)–(f) Radial strain anterior and inferior segments. (a)-(b) Radial strain apex, inferior and anterior segments per group. Strain in the anterior section of the LAD study is significantly lower during follow-up than at baseline. (c)-(d) Radial strain mid-ventricular level, inferior and anterior segments per group. (e)-(f) Radial strain basal level, inferior and anterior segments per group. (g)–(j) Circumferential strain. (g) Apical circumferential strain over time. (h) Circular strain at mid-ventricular level over time. (i) Circumferential strain at basal level over time. (j) Total ventricular circumferential strain over time. LCx indicates left circumflex artery. LAD: left anterior descending artery; BL: baseline. ^*^ *P* < 0.05 within the LAD group.

Decreased strain indicates deteriorated contractile function of the myocardium. In the LCx group, no changes in radial strain were observed in the infarcted inferior segment (Figure 4).

For the LAD group, radial strain was significantly attenuated in the anterior segment of the apex during FU and kept progressively declining (42.8 ± 4.8% at baseline and 5.6 ± 7.7% at 12 weeks of FU; *P* = 0.008; Figure 4). In this group, total apical radial strain at 12 weeks of FU also decreased to 20.0 ± 4.5% as opposed to 45.1 ± 4.1% at baseline (*P* = 0.04). The total ventricular strain at 12 weeks of FU was significantly lower in this group at 12 weeks of FU (48.6 ± 2.0% at baseline and 30.1 ± 3.8% at follow-up; *P* = 0.03). These findings corroborate with the LVEF echocardiography data. Circumferential strain was significantly reduced in the LAD group in the apex and at mid-ventricular level of the heart throughout the 12 weeks of FU compared to baseline (Figure 4).

### 4.4. PV Loop Analysis, Infarct Size, Troponin, and Histology

**Figure 5:**
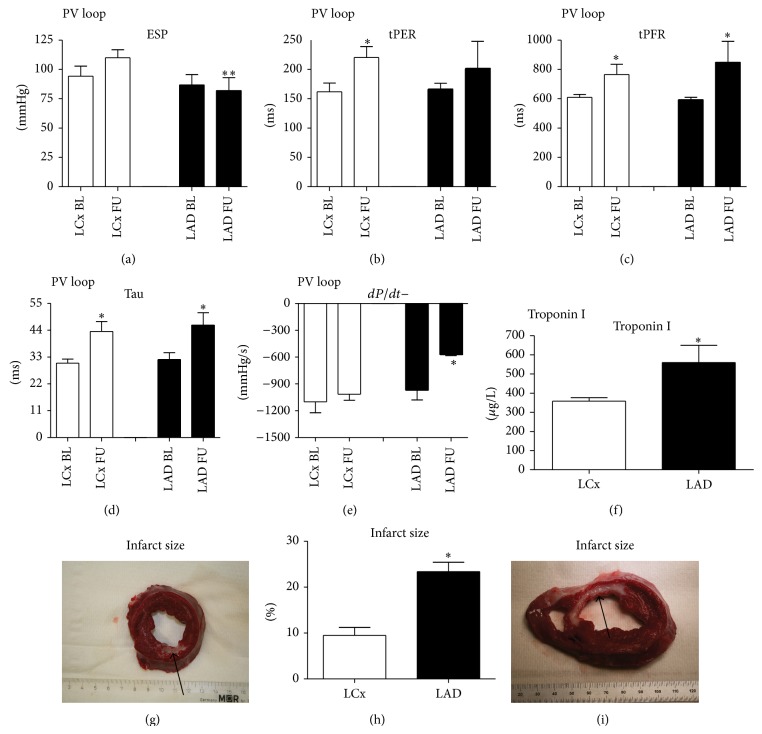
Pressure-volume loop analysis, troponin I, and infarcts size. (a)–(e) Pressure-volume loop analysis. (a) End-systolic pressure (ESP) is significantly lower at follow-up in the LAD group; (b) time to peak ejection rate (tPER); (c) time to peak filling rate (tPFR); (d) Tau: relaxation constant; (e) increase in pressure over time (*dP*/*dt*+); (f) troponin I levels 6 hours after myocardial infarction are higher in the LAD group. (g)–(i) Infarct size. (g) Example of infarct by left circumflex artery infarct (LCx) ischemia-reperfusion model. Viable myocardium is red; infarct is depicted in white (arrow). (h) Infarct size in left anterior descending artery (LAD) group is significantly higher; (i) example of an LAD infarct. LCx indicates left circumflex artery. LAD: left descending anterior artery; BL: baseline; FU: follow-up. ^*^ *P* < 0.05.

Invasive real-time PV loop analysis was also performed at baseline and 12 weeks of FU. *dP*/*dt*− (relaxation of the ventricle) and ESPVR (derivative of contractility) were significantly decreased in the LAD group but not in the LCx group (Figure 5). Indices of diastolic dysfunction, Tau and tPFR (time to peak filling rate), and systolic indices tPER (time to peak ejection rate) increased in both groups. Troponin I levels 6 hours after infarct induction in the LCx group were lower than in the LAD group (358.6 ± 79.9 *μ*g/L versus 560.0 ± 79.9 *μ*g/L, *P* = 0.02; Figure 5). Post-AMI decrease in cardiac function and troponin levels 6 hours after infarct correlated significantly (*R* = −0.763; *P* = 0.028). The average infarct size in the LAD group was significantly higher than in the LCx group (23.4 ± 2.1% in the LAD group versus 9.5 ± 1.7% in the LCx group, Figure 5). There were no differences in collagen density, capillary density, and arteriole density in all myocardial segments (Table 2). A trend towards an increase in arteriole density was observed in the LAD group (*P* = 0.09).

## 5. Discussion

The potential effects of novel therapeutics that confine the damage after MI depend on the severity of the ischemic event [3]. To determine which patient group (e.g., moderately or severely affected patients) benefits most from such therapeutics, their efficacy has to be tested in large animal models that resemble the clinical course and severity as closely as possible [4, 6, 21]. Therefore, the current study investigated cardiac function over time in two clinically relevant porcine models of ischemia-reperfusion: a severe LAD model and a moderate LCx ischemia-reperfusion model. Here, we demonstrated that occlusion of 150 minutes of the LAD resulted in overt deterioration of cardiac function and progressive cardiac dilatation. Therefore, this model is most suitable for testing therapeutics that focus on the prevention of adverse remodeling after MI in severely affected patients. On the other hand, occlusion of the LCx resulted in limited effects on cardiac contractility and dilatation, resembling the clinical course of adequately revascularized MI patients with only limited cardiac damage. Thus, this model can be used to test compounds that reduce infarct size directly after MI but is less suitable for prevention of adverse remodeling.

As hypothesized, the two models showed a marked difference in response to ischemia-reperfusion. First, in the LCx model, none of the animals died which was, most likely, based on fewer episodes of VF. Presumably this is due to the involvement of the septum in the infarct area in the LAD model combined with a smaller area at risk after occlusion of the LCx. This difference in the area at risk also resulted in a large difference in infarct size, which was approximately 23% of the LV in the LAD model compared to 10% in the LCx model. In turn, the larger infarct size in the LAD model resulted in a profound decrease in LVEF culminating in adverse remodeling and cardiac dilatation. The decrease in LVEF occurred to a lesser extent in the LCx group and this did not result in any cardiac dilatation after MI. In concordance with the functional echocardiography data, myocardial strain was decreased in the affected myocardial segments in the LAD group, whereas strain was preserved in the affected segments in the LCx group. To the best of our knowledge, this is the first study that has studied myocardial strain when comparing different MI models and these findings show that the two models are not only different regarding the global cardiac function but also differ on a regional contractile level. Despite the significant differences in systolic function in the LAD group, no differences between the groups were observed regarding diastolic dysfunction. In both studies, diastolic dysfunction worsened relative to baseline measures, which suggests that these measurements are very robust and are not directly influenced by infarct size or dilatation in the current study.

In a previous study of Suzuki et al. in which the mid-LAD was occluded for 60 minutes followed by reperfusion, a comparable mortality (16%) and decrease in cardiac function were observed following infarct induction [18]. However, in that study LVEF decreased transiently and recovered significantly at 14 days and 28 days of follow-up to 47%. This is most likely due to the occlusion time. A 60-minute occlusion period presumably leads to reversible cell damage, with a large fraction of myocardial stunning/hibernation that is known to resolve spontaneously within days to weeks [30]. In our study, LVEF did not recover during FU after the initial decrease after MI in either model. In the LAD model this simultaneously occurred with increased LVESV and LVEDV, suggesting progression into heart failure. This provides a larger therapeutic window for experimental therapies that target adverse remodeling and prevent cardiac dilatation in this model compared to spontaneously recovering models. The same holds true for the LCx model. Again, therapeutic efficacy is easier to test in a model in which outcome is not affected by confounding factors such as spontaneous recovery that may exceed and hence mask the therapeutic effect. Importantly, our findings in both the LAD model and the LCx model on cardiac function and infarct size correspond to chronic myocardial ischemia models [15, 16]. This indicates that 150-minute occlusion followed by reperfusion results in the same amount of myocardial damage but more closely resembles the clinical course and treatment of MI. Moreover, in our models, intracoronary therapy to limit myocardial damage is still possible.

Despite our best efforts, the study has some limitations. First, we assessed cardiac function by 2D echocardiography during follow-up and obtained 3D echocardiogram directly after MI (LAD-model only) and at sacrifice (both models). Due to the anatomical position of porcine ribs, it is not possible to obtain a transthoracic 4 chamber view that would be needed for 3D echocardiography. Although cardiac MRI is considered the gold standard for cardiac function and volumes, due to logistic reasons, it was not feasible to obtain sequential cardiac MRI data in our study. We are confident that our echocardiography data are reproducible and depict the true cardiac function following both ischemia-reperfusion models. Second, in one group we performed a closed chest balloon occlusion model and in the other group an open chest model was applied. The latter was chosen in order to be able to perform epicardial defibrillation in the LAD model to improve survival in the LAD model. Since the study was not designed to directly compare these two models, a similar infarct induction was, however, not essential.

To conclude, the current study showed that it is feasible to create two very distinctive models of chronic myocardial infarction to test novel therapeutics after MI for the possible treatment of different patient groups. Reperfusion after 150 min ischemia of the LAD leads to severe cardiac dysfunction and the development of heart failure over a limited period of time, whereas ischemia-reperfusion of the LCx culminates in stable, moderate cardiac dysfunction. Since these models closely resemble the clinical course and treatment of MI and enable intracoronary therapy administration, they are preferable to models with persistent coronary occlusion. This study adds to refinement of preclinical studies which hopefully will result in improved translational medicine and a reduction of animals needed in preclinical research.

## Figures and Tables

**Table 1:** Echocardiography data.

Echocardiography	LCx	*P* value to BL^*^	LAD	*P* value to BL^*^	*P* value between groups^#^
LVEF baseline	56.7 ± 2.0		56.4 ± 1.5		NS
LVEDV baseline	80.8 ± 6.6		68.3 ± 3.7		NS
LVESV baseline	35.3 ± 4.3		30.1 ± 4.0		NS

LVEF PMI	50.8 ± 1.6	0.03	39.4 ± 1.6	0.009	0.02
LVEDV PMI	83.7 ± 4.3	NS	79.2 ± 5.8	NS	NS
LVESV PMI	40.9 ± 1.1	NS	47.9 ± 3.7	0.01	0.09

LVEF 4 weeks	48.6 ± 1.0	0.02	40.3 ± 0.7	<0.001	0.04
LVEDV 4 weeks	88.6 ± 4.4	NS	96.5 ± 4.3	0.01	0.06
LVESV 4 weeks	45.5 ± 2.0	0.07	57.7 ± 2.8	0.01	0.03

LVEF 8 weeks	51.4 ± 1.0	NS	39.9 ± 1.0	<0.001	0.04
LVEDV 8 weeks	95.8 ± 5.9	NS	97.4 ± 7.8	0.06	NS
LVESV 8 weeks	46.5 ± 3.4	0.06	58.5 ± 5.0	0.01	0.07

LVEF 12 weeks	49.3 ± 0.8	0.06	41.7 ± 1.5	<0.001	0.004
LVEDV 12 weeks	91.4 ± 4.9	NS	104.1 ± 5.7	0.02	0.03
LVESV 12 weeks	46.0 ± 2.4	0.06	60.7 ± 4.1	<0.001	0.04

Table 1 represents all data and *P* values for echocardiography. The postmyocardial infarct values of the LAD study are measured with 3D echocardiography. LCx indicates left circumflex artery. LAD: left anterior descending artery; LVEF: left ventricular ejection fraction; LVEDV: left ventricular end-diastolic volume; LVESV: left ventricular end-systolic volume. BL: baseline before infarct, PMI: post myocardial infarct. ^*^Repeated measures ANOVA (values compared to baseline), ^#^Student's *t*-test.

**Table 2:** Histological analysis.

Parameter	Measure	LCx	LAD	*P* value
Collagen density infarct area	%	89.2 ± 4.0	89.2 ± 4.2	NS
Collagen density border area	%	5.1 ± 1.5	5.5 ± 1.3	NS
Collagen density remote area	%	4.4 ± 1.1	3.0 ± 0.7	NS
Arteriole density infarct area	Arterioles/view	11.2 ± 1.5	10.1 ± 1.9	NS
Arteriole density border area	Arterioles/view	5.1 ± 1.1	9.2 ± 1.4	NS
Arteriole density remote area	Arterioles/view	3.5 ± 0.5	5.4 ± 0.9	NS
Capillary density border area	Capillaries/mm²	713.9 ± 74.3	714.4 ± 85.5	NS
Capillary density remote area	Capillaries/mm²	470.5 ± 48.0	376.4 ± 37.9	NS

Overview of histological parameters. NS indicates nonsignificant difference between the groups.
